# The reliability and validity of the TBI-CareQOL system in four diverse caregiver groups

**DOI:** 10.1186/s41687-023-00602-x

**Published:** 2023-06-26

**Authors:** Noelle E. Carlozzi, Sung Won Choi, Zhenke Wu, Srijan Sen, Jonathan Troost, Angela K. Lyden, Jennifer A. Miner, Christopher Graves, Angelle M. Sander

**Affiliations:** 1 Department of Physical Medicine and Rehabilitation, University of Michigan, North Campus Research Complex, 2800 Plymouth Road, Building NCRC B14, Room G216, Ann Arbor, MI 48109-2800 USA; 2 Department of Pediatrics, University of Michigan, Ann Arbor, MI USA; 3 Department of Biostatistics, School of Public Health, University of Michigan, Ann Arbor, MI USA; 4 Michigan Institute for Data Science, University of Michigan, Ann Arbor, MI USA; 5 Department of Psychiatry, University of Michigan, Ann Arbor, MI USA; 6 Clinical Trials Support Office, University of Michigan, Ann Arbor, MI USA; 7 H. Ben Taub Department of Physical Medicine and Rehabilitation, Baylor College of Medicine/Harris Health System, Houston, TX USA; 8 Brain Injury Research Center, TIRR Memorial Hermann, Houston, TX USA

**Keywords:** Quality of life, Informal caregivers, Caregiver burden, Patient-reported outcome, Psychometrics

## Abstract

**Purpose:**

Establishing the psychometric reliability and validity of new measures is an ongoing process. More work is needed in to confirm the clinical utility of the TBI-CareQOL measurement development system in both an independent cohort of caregivers of traumatic brain injury (TBI), as well as in additional caregiver groups.

**Methods:**

An independent cohort of caregivers of people with TBI (n = 139), as well as three new diverse caregiver cohorts (n = 19 caregivers of persons with spinal cord injury, n = 21 caregivers for persons with Huntington disease, and n = 30 caregivers for persons with cancer), completed 11 TBI-CareQOL measures (caregiver strain; caregiver-specific anxiety; anxiety; depression; anger; self-efficacy; positive affect and well-being; perceived stress; satisfaction with social roles and activities; fatigue; sleep-related impairment), as well as two additional measures to examine convergent and discriminant validity (PROMIS Global Health; the Caregiver Appraisal Scale).

**Results:**

Findings support the internal consistency reliability (all alphas > 0.70 with the vast majority being > 0.80 across the different cohorts) of the TBI-CareQOL measures. All measures were free of ceiling effects, and the vast majority were also free of floor effects. Convergent validity was supported by moderate to high correlations between the TBI-CareQOL and related measures, while discriminant validity was supported by low correlations between the TBI-CareQOL measures and unrelated constructs.

**Conclusion:**

Findings indicate that the TBI-CareQOL measures have clinical utility in caregivers of people with TBI, as well as in other caregiver groups. As such, these measures should be considered as important outcome measures for clinical trials aiming to improve caregiver outcomes.

## Abstract (highlights)

As the complexities of disease management and care needs grow, so does the need for informal caregivers. Providing care for a family member or friend with a significant health condition can have a deleterious impact on caregiver health-related quality of life (HRQOL), as well as the HRQOL of the care recipient. Reliable and valid measures of HRQOL can help us to better understand the treatment needs of these caregiver populations.

## Introduction

The prevention and treatment of health conditions have traditionally been placed on the individual patient. However, illness impacts the entire family, and the complete picture of health conditions is a collage of the affected patient and family caregiver, experienced by both. While significant scientific discoveries and advances in human health are being made, the complexities of health management and care needs are also growing substantially. As a society, these needs have always been placed on family caregivers who eventually face enormous burden, providing care to a loved one, while maintaining their own health and well-being (e.g., health-related quality of life, HRQOL). It is well-established that the burden of caregiving adversely affects mental well-being, and further prolonged caregiving may even worsen physical health [1–34]. In turn, caregiver HRQOL can also have a profound impact on care-recipient HRQOL [12, 24–31, 35–43]. For decades, the nation’s rapidly aging population and increasing need for caregiving have been called to attention. However, very little action has been taken and this remains a major overlooked challenge facing the aging U.S. population. Thus, family caregiving is an urgent public health issue.

Until recently, existing clinical trials to improve caregiver and care recipient HRQOL was stymied by a lack of available measures to comprehensively assess the multiple areas of HRQOL that are impacted by the caregiver role. To address this gap in measurement, the TBI-CareQOL measurement system was developed to capture important HRQOL concepts specific to caregivers of people with traumatic brain injury [44–52]. This system includes 27 item banks that represent aspects of HRQOL that are applicable across different diseases and conditions, as well as those aspects that are more unique to caregivers or caregivers of people with traumatic brain injury, specifically. Preliminary data in the development cohorts has supported the clinical utility of this measurement system in caregivers of people with TBI, but it has yet to be confirmed in an independent sample of caregivers.

In addition, most of the concepts that the TBI-CareQOL captures, at least at face value, are broadly applicable to other caregiving populations. We would expect that the clinical utility of these measures should be broadly applicable to caregivers of people that experience other adult-onset traumatic injuries. We would also like to determine if these measures will demonstrate clinical utility in caregiver cohorts of individuals caring for those with insidious, progressive conditions, as well as caregiver cohorts of individuals dealing with more episodic relapsing and remitting conditions. As such, we need to examine the psychometric properties of these new measures in diverse caregiver populations, in order to establish their clinical utility as primary or secondary endpoints in clinical trials.

Therefore, the purpose of this analysis was to provide data to support the psychometric reliability and validity of 11 of the TBI-CareQOL measurement system item banks (caregiver strain, caregiver-specific anxiety, anxiety, depression, anger, self-efficacy, positive affect and well-being, perceived stress, satisfaction with social roles and activities, fatigue and sleep-related impairment) in an independent sample of caregivers of people with TBI, as well as in three new diverse caregiver cohorts (caregivers of persons with: (1) a chronic condition that was caused by a traumatic event [SCI = spinal cord injury], (2) caregivers for persons with a progressive, fatal neurodegenerative disease [HD = Huntington disease], and (3) caregivers for persons with an episodic cancer condition that requires intense, prolonged inpatient and outpatient treatment (HCT = allogeneic hematopoietic cell transplantation]). We provide data examining the internal consistency, measurement characteristics, concurrent validity, and discriminant validity of these 11 TBI-CareQOL system item banks across these four diverse cohorts to contribute to a body of growing research to support both their clinical utility in caregivers of people with TBI, but also in other, independent caregiver cohorts.

## Method

### Participants

The data examined in this analysis represents data collected across two different studies: an independent cohort of N = 139 caregivers of people with TBI (NCT04570930), as well as three new caregiver cohorts: N = 19 caregivers of people with SCI, N = 21 caregivers of people with HD, and N = 30 caregivers of people undergoing HCT (NCT04556591). The detailed protocols for these RCTs are published and available for review [53, 54]. Data collection for the TBI cohort occurred at both the University of Michigan and Baylor College of Medicine/TIRR Memorial Hermann; data collection for the SCI, HD, and HCT cohorts occurred only at the University of Michigan. Several recruitment sources were used including hospital and community-based recruitment, as well as lab-specific research registries (of both caregivers and patients), medical record data capture systems [55], and a website and social media postings. Caregivers were recruited directly, or through the person for whom they provide care (i.e., the individual with TBI, SCI, HD or HCT).

All caregivers had to be at least 18 years of age, able to read and understand English, and caring for an adult (i.e., ages 18 and above) with a medically documented SCI, HD, HCT, or TBI. Caregivers had to indicate a response ≥ 1 on the following question: “On a scale of 0–10, where 0 is ‘no assistance’ and 10 is ‘assistance with all activities’, how much assistance does the person you care for require from you to complete activities of daily living due to problems resulting from his/her TBI/HD/SCI/HCT? Activities could consist of personal hygiene, dressing and undressing, housework, taking medications, managing money, running errands, shopping for groceries or clothing, transportation, meal preparation and cleanup, remembering things, etc.” In addition, caregivers of individuals with SCI and TBI had to be caring for someone that was ≥ 1 year post-injury and caregivers of persons with HCT had to be caring for an individual who was receiving, had received or was scheduled to receive HCT. Caregivers also had to be willing to use their own technology (i.e., smartphone/tablet and internet access) and be willing to download the CareQOL app and the Fitbit^®^ app on their personal device. Paid, professional caregivers were excluded.

### Study design

Both studies used a two-arm randomized controlled design. Study participation involved a 2-h baseline virtual study visit followed by a 10-day run-in period then a 6-month (120 day) or 3-month (90 day) home monitoring period (for the TBI study, and the SCI/HCT/HD study, respectively). Data for this analysis was from the baseline visit (i.e., the 2-h virtual study visit; data collection occurred from December 2020 through February 2022 for the TBI study, and from November 2020 through March 2021 for the SCI/HCT/HD study) which involved informed consent, the completion of several self-report measures and instructions for the home-monitoring period. All study procedures were approved by the institutional review board (IRBs) for the data collection sites (IRBMED HUM00184455, HUM00181282, and HUM00186921; IRBMED Baylor College of Medicine SITE0000087; Baylor College of Medicine/Memorial Hermann IRB number H-48478). These trials are also registered with ClinicalTrials.gov (NCT04556591 and NCT04570930).

### Measures

The measures that were considered for these analyses are reported below.

*TBI-CareQOL Caregiver-specific measures*. Two caregiver-specific measures were administered from the TBI-CareQOL measurement system: (1) TBI-CareQOL Caregiver Strain short form (SF) [44, 56] assesses perceived feelings of feeling overwhelmed, stressed and “beat-down” related to the caregiver role; and (2) TBI-CareQOL Caregiver-Specific Anxiety SF [44, 57] assesses perceived feelings of worry and anxiety specific to the safety, health, and future well-being of the person with TBI. Items on these measures that referred to the “person with the injury” were modified to read “person with the illness” for use in the HD and HCT cohort. These measures are scored on a *T* metric (M = 50; SD = 10). Higher scores indicate more strain or caregiver-specific anxiety, respectively. Administration times for each of these measures is ~ 1 min.

*TBI-CareQOL Mental Health Measures*. Six caregiver-specific measures were administered from the TBI-CareQOL measurement system: (1) TBI-CareQOL PROMIS Anxiety SF [58] assesses self-reported feelings of fear, anxiety and hyperarousal; (2) TBI-CareQOL PROMIS Depression SF [58] assesses self-reported feelings of sadness and worthlessness; (3) TBI-CareQOL PROMIS Anger SF [58] assesses self-reported feelings of irritability and frustration; (4) TBI-CareQOL NIH Toolbox Self-Efficacy [59] assesses self-reported confidence in the ability to successfully perform specific tasks or behaviors related to one’s overall functioning; (5) TBI-CareQOL Neuro-QoL Positive Affect & Well-Being SF [60] assesses parts of an individual’s life that are related to overall life meaning and purpose, well-being and satisfaction; and (6) TBI-CareQOL NIH Toolbox Perceived Stress SF [59] assesses an individual’s feelings about the nature of events and individual coping resources. These measures are all scored on a *T* metric (M = 50; SD = 10). Higher scores indicate more of the named construct (i.e., worse HRQOL for negatively named constructs and better HRQOL for positively named constructs). Administration time for each measure is ~ 1 min.

*TBI-CareQOL Social Health Measure*. The PROMIS Ability to Participate in Social Roles and Activities SF [58] assesses involvement in one’s ability to participate in usual social roles and activities. This measure is scored on a *T* metric (M = 50; SD = 10). Higher scores indicate more ability to participate. Administration time is ~ 1 min.

*TBI-CareQOL Physical Health Measures*. Two TBI-CareQOL physical health measures were administered: (1) TBI-CareQOL PROMIS Sleep-Related Impairment SF[61] assesses the effect of poor sleep on daytime functioning; and (2) TBI-CareQOL PROMIS Fatigue SF [58] assesses self-reported symptoms of fatigue, ranging from mild subjective feelings of tiredness to overwhelming exhaustion that may decrease one’s ability to perform activities of daily living. These measures are also scored on a *T* metric (M = 50; SD = 10). Higher scores indicate more sleep-related impairment or fatigue, respectively. Administration time for each of these measures is ~ 1 min.

#### Convergent and discriminant validity measures

PROMIS Global Health v1.2 [62] is a 10-item patient-reported outcome measure that assesses overall physical, mental, and social health. This measure is scored on a *T* metric (M = 50; SD = 10); separate scores are generated for physical and mental health. Higher scores indicate better health. The administration time for this measure is ~ 3 min. Mental health scores were used to demonstrate convergent validity for 4 of the 11 TBI-CareQOL measures (Anxiety, Depression, Anger, and Positive Affect and Well-Being). Physical health scores were used to demonstrate convergent validity for 2 of the 11 TBI-CareQOL measures (Fatigue and Sleep-related Impairment). PROMIS Global Health has been used in different caregiver populations and has data to support its clinical utility in these cohorts [63–65].

The Caregiver Appraisal Scale [66] is a 47-item measure that is designed to capture the positive and negative aspects of caregiving. Sum scores are calculated to reflect four separate subdomain scores (perceived burden, caregiver relationship satisfaction, caregiving ideology, and caregiving mastery); higher scores indicating better functioning. This measure takes between 5 and 10 min to complete. Items and instructions for this measure that referred to the “person with the injury” were modified to read “person with the illness” for use in the HD and HCT cohort. Subdomain scores on Caregiver Burden were used to demonstrate convergent validity for 4 of the 11 TBI-CareQOL measures (Caregiver-Specific Anxiety, Caregiver Strain, Perceived Stress, and Ability to Participate in Social Roles and Activities) and subdomain scores on Ideology and Mastery were used to demonstrate discriminant validity of the CareQOL measures. The Caregiver Appraisal Scale has been used in different caregiver populations and has data to support the reliability and validity across these cohorts [66–69].

### Statistical analyses

Analyses were conducted using SAS v9.4; analyses were run separately for each of the four caregiver groups, as well as for the combined sample.

*Internal Consistency and Measurement Characteristics*. Internal consistency reliability was examined using Cronbach’s alpha. Minimal acceptable reliability was specified as ≥ 0.70 [70, 71]. Measurement characteristics for floor and ceiling effects were also calculated (i.e., the percentage of participants who had the highest or lowest possible SF score respectively). The criterion for floor and ceiling rates was ≤ 20% [72, 73].

*Convergent and Discriminant Validity*. Pearson correlations among the different measures were used to examine convergent and discriminant validity. Convergent validity would be supported by moderate to strong correlations (“moderate” = *r*’s ≥ 0.36—0.67 and “high” = *r’s* between 0.68 and 0.89) between each TBI-CareQOL measure and its hypothesized comparator [74]. Discriminant validity would be supported by weak correlations (“low” = *r*’s ≤ 0.35) between each TBI-CareQOL measure and measures of caregiver mastery and caregiver ideology from the Caregiver Appraisal Scale [74].

## Results

A total of 139 caregivers of persons with TBI, as well as 19 caregivers of persons with SCI, 21 caregivers of persons with HD, and 30 caregivers of persons with HCT were enrolled in this study. The sample characteristics are provided in Table 1.

**Table 1:** Sample characteristics

Variable	HD	SCI	HCT	TBI
	(n = 21)	(n = 19)	(n = 30)	(n = 139)
*Sex (%)*				
Female	62	74	73	78
Male	38	26	23	22
Not reported	0	0	3	0
*Race (%)*				
American Indian or Alaska Native (including all Original Peoples of the Americas)	0	0	0	1
Asian	0	11	3	3
Black or African American (including Africa, Caribbean)	0	11	0	17
White or Caucasian (including Middle Eastern)	100	74	90	73
More than one race	0	5	7	5
Missing	0	0	0	1
*Ethnicity (%)*				
Non-Hispanic	90	95	100	83
Hispanic	10	0	0	17
Missing	0	5	0	0
*Marital status (%)*				
Married or Cohabitating	81	89	80	76
Not married but has significant other	0	5	0	6
Single and divorced	14	0	10	9
Single and never married	0	5	10	7
Single and widowed	0	0	0	1
Missing	5	0	0	0
*Work status (%)*				
Full-time (at least 40 h per week)	52	42	43	48
Part-time	5	26	7	11
Homemaker	0	0	3	9
Student	0	0	3	4
Retired	43	16	27	20
Disabled	0	0	0	4
Unemployed	0	16	17	4
*Age (years)*				
M (SD)	60.8 (9.39)	50.4 (15.09)	54.4 (14.33)	51.2 (15.35)
*Time in caregiver role (years)*				
M (SD)	9.0 (7.69)	13.0 (7.6)	2.4 (3.6)	7.2 (5.76)
*Relationship to patient (%)*				
Partner/Spouse	71	42	63	45
Child	0	21	3	12
Parent	14	11	20	28
Sibling	10	5	10	10
Other family	0	0	0	1
In-law	0	5	0	0
Friend	5	16	3	1
Missing	0	0	0	1
*Time caregiving (%)*				
1-2 h/day or less	48	21	33	55
3-4 h/day (half a working day)	38	32	37	21
5-8 h/day (full working day)	10	26	10	10
9-12 h/day	0	0	7	4
> 12 h/day or round-the-clock care	5	21	10	10
Missing	0	0	3	0
*Age of care-recipient (years)*				
M (SD)	57.9 (9.61)	48.1 (14.97)	54.1 (16.31)	43.3 (16.54)
*Sex of care-recipient (%)*				
Female	48	37	27	15
Male	52	63	73	85
*UHDRS independence scale (%)*				
30: minimal assistance in own feeding, bathing, toileting	0	0	3	–
40: chronic care facility needed; limited self-feeding, liquified diet	0	0	0	–
50: 24-h supervision appropriate; assistance required for bathing, eating, toileting	24	63	0	–
60: minor assistance in dressing, toileting, bathing; food must be cut for subject	14	21	7	–
70: self-care maintained for bathing, limited household duties (cooking and use of knives), driving terminates; unable to manage finances	14	5	27	–
80: pre-disease level of employment changes or ends; cannot perform household chores to pre-disease level, may need help with finances	10	5	23	–
90: no physical care needed if difficult tasks are avoided	19	5	23	–
100: no special care needed	19	0	13	–
Missing	0	0	3	–

*Overall, HCT caregivers reported significant less years in their role than the other two cohorts (*p* < 0.002); there were no other significant differences among these four groups for these demographic variables

For the 11 TBI-CareQOL HRQOL measures, internal consistency reliability ranged from acceptable to excellent for the SCI and HCT samples, and from good to excellent for the HD and TBI samples; in all cases the a priori criterion for acceptable reliability was met (Table 2). There were no ceiling effects for any of the TBI-CareQOL measures for any of the cohorts (Table 2). There was a floor effect for Depression for all cohorts except TBI; there was also a slight floor effect for Caregiver-Specific Anxiety and Anxiety for the SCI sample.

**Table 2:** Descriptive information and reliability data for TBI-CareQOL measures

	M (SD)	Internal consistency	Floor %	Ceiling %
Caregivers of persons with spinal cord injury (*n* = 19)				
*Caregiver-specific HRQOL*				
TBI-CareQOL caregiver-specific anxiety	44.4 (7.92)	0.90	21.05	0.00
TBI-CareQOL caregiver strain	49.3 (7.62)	0.88	0.00	0.00
*Mental HRQOL*				
Neuro-QoL anxiety SF	50.2 (8.75)	0.84	36.84	0.00
Neuro-QoL depression SF	49.2 (7.91)	0.74	42.11	0.00
PROMIS anger SF	50.8 (10.59)	0.94	0.00	0.00
Neuro-QoL positive affect and well being SF	54.2 (6.17)	0.92	0.00	5.26
NIH toolbox self-efficacy SF	47.2 (5.71)	0.76	0.00	0.00
NIH toolbox perceived stress SF	51.6 (11.44)	0.88	0.00	0.00
*Social HRQOL*				
Neuro-QoL ability to participate in SRA SF	47.2 (10.24)	0.95	5.26	15.79
*Physical HRQOL*				
PROMIS sleep related impairment SF	54.1 (8.39)	0.91	0.00	0.00
PROMIS fatigue SF	51.3 (9.96)	0.95	10.53	0.00
Caregivers of Persons with Huntington Disease (*n* = 21)				
*Caregiver-specific HRQOL*				
TBI-CareQOL caregiver-specific anxiety	51.4 (7.19)	0.90	0.00	0.00
TBI-CareQOL caregiver strain	50.4 (7.02)	0.89	0.00	0.00
*Mental HRQOL*				
Neuro-QoL anxiety SF	54.5 (8.03)	0.92	14.29	0.00
Neuro-QoL depression SF	51.4 (9.96)	0.92	38.10	0.00
PROMIS anger SF	51.3 (12.02)	0.95	0.00	0.00
Neuro-QoL positive affect and well being SF	51.9 (7.07)	0.97	0.00	0.00
NIH toolbox self-efficacy SF	48.0 (8.75)	0.94	0.00	0.00
NIH toolbox perceived stress SF	46.1 (13.49)	0.93	0.00	0.00
*Social HRQOL*				
Neuro-QoL ability to participate in SRA SF	49.6 (7.61)	0.88	0.00	9.52
*Physical HRQOL*				
PROMIS sleep related impairment SF	51.4 (8.34)	0.93	0.00	0.00
PROMIS fatigue SF	49.0 (9.05)	0.94	4.76	0.00
Caregivers of persons with HCT (*n* = 30)				
*Caregiver-specific HRQOL*				
TBI-CareQOL caregiver-specific anxiety	47.2 (5.86)	0.78	3.33	0.00
TBI-CareQOL caregiver strain	47.6 (7.12)	0.84	3.33	0.00
*Mental HRQOL*				
Neuro-QoL anxiety SF	53.8 (8.25)	0.88	16.67	0.00
Neuro-QoL depression SF	50.1 (7.40)	0.76	33.33	0.00
PROMIS anger SF	49.6 (8.99)	0.89	0.00	0.00
Neuro-QoL positive affect and well being SF	53.8 (5.16)	0.93	0.00	0.00
NIH toolbox self-efficacy SF	50.4 (9.01)	0.90	0.00	6.67
NIH toolbox perceived stress SF	49.5 (10.22)	0.90	0.00	0.00
*Social HRQOL*				
Neuro-QoL ability to participate in SRA SF	46.0 (7.78)	0.90	3.33	3.33
*Physical HRQOL*				
PROMIS sleep related impairment SF	51.2 (10.97)	0.93	10.00	0.00
PROMIS fatigue SF	50.4 (10.38)	0.94	3.33	3.33
Caregivers of persons with traumatic brain injury (*n* = 139)				
*Caregiver-specific HRQOL*				
TBI-CareQOL caregiver-specific anxiety	51.2 (9.18)	0.92	7.19	0.00
TBI-CareQOL caregiver strain	50.0 (8.69)	0.88	3.60	0.72
*Mental HRQOL*				
Neuro-QoL anxiety SF	57.1 (7.80)	0.86	7.91	0.72
Neuro-QoL depression SF	53.3 (7.27)	0.84	15.83	0.00
PROMIS anger SF	52.0 (9.47)	0.91	0.00	0.00
Neuro-QoL positive affect and well being SF	52.9 (5.81)	0.90	0.00	1.44
NIH toolbox self-efficacy SF	47.2 (8.90)	0.91	0.00	1.44
NIH toolbox perceived stress SF	54.0 (10.53)	0.89	0.00	0.00
*Social HRQOL*				
Neuro-QoL ability to participate in SRA SF	47.0 (8.43)	0.91	2.16	8.63
*Physical HRQOL*				
PROMIS sleep related impairment SF	54.9 (9.28)	0.92	2.16	0.72
PROMIS fatigue SF	52.4 (9.97)	0.94	7.19	4.32

*SRA* social roles and activities

For all of the measures and across all of the samples, a priori hypotheses for convergent validity were met (see Table 3). With regard to discriminant validity (Table 3), the majority of findings were consistent with our hypothesized expectations. Exceptions included stronger relationships between Caregiver-Specific Anxiety and Caregiver Ideology for the SCI and HCT samples, between Strain and Caregiver Ideology for the HD sample, between Positive Affect and Well-being and Caregiver Mastery for the HD sample, the two physical function measures (Sleep-related Impairment and Fatigue) and Caregiver Mastery for the HD sample, and between Positive Affect and Well-Being and Caregiver Ideology for the HCT sample.

**Table 3:** Convergent and discriminant validity

HRQOL measure	Convergent validity	Discriminant validity	
		CAS ideology	CAS mastery
Caregivers of persons with spinal cord injury (n = 19)			
*Caregiver-specific HRQOL*			
TBI-CareQOL caregiver-specific anxiety	CAS Burden − 0.82*	0.47*	− 0.11
TBI-CareQOL caregiver strain	CAS Burden − 0.78*	0.20	− 0.01
*Mental HRQOL*			
Neuro-QoL anxiety SF	PROMIS GLOBAL Mental Health − 0.46*	0.23	− 0.17
Neuro-QoL depression SF	PROMIS GLOBAL Mental Health − 0.70*	0.17	− 0.18
PROMIS anger SF	PROMIS GLOBAL Mental Health − 0.71*	0.20	− 0.24
Neuro-QoL positive affect and well being SF	PROMIS GLOBAL Mental Health 0.78*	− 0.24	0.07
NIH toolbox self-efficacy SF	Neuro-QoL Positive Affect and Well-Being 0.72*	− 0.22	− 0.08
NIH toolbox perceived stress SF	CAS Burden − 0.66	0.10	− 0.19
*Social HRQOL*			
Neuro-QoL ability to participate in SRA SF	CAS Burden 0.62*	− 0.13	0.13
*Physical HRQOL*			
PROMIS sleep related impairment SF	PROMIS GLOBAL Physical Health − 0.54*	− 0.01	− 0.10
PROMIS fatigue SF	PROMIS GLOBAL Physical Health − 0.70*	0.12	− 0.12
Caregivers of persons with Huntington disease (*n* = 21)			
*Caregiver-specific HRQOL*			
TBI-CareQOL caregiver-specific anxiety	CAS Burden − 0.66*	0.12	− 0.31
TBI-CareQOL caregiver strain	CAS Burden − 0.78*	0.41	− 0.29
*Mental HRQOL*			
Neuro-QoL anxiety SF	PROMIS GLOBAL Mental Health − 0.81*	0.24	− 0.16
Neuro-QoL depression SF	PROMIS GLOBAL Mental Health − 0.92*	0.24	− 0.19
PROMIS anger SF	PROMIS GLOBAL Mental Health − 0.80*	0.13	− 0.18
Neuro-QoL Positive affect and well being SF	PROMIS GLOBAL Mental Health 0.85*	− 0.005	0.37
NIH toolbox self-efficacy SF	Neuro-QoL Positive Affect and Well-Being 0.69*	0.22	0.34
NIH toolbox perceived stress SF	CAS Burden − 0.82*	0.13	− 0.31
*Social HRQOL*			
Neuro-QoL ability to participate in SRA SF	CAS Burden 0.66*	− 0.34	0.14
*Physical HRQOL*			
PROMIS sleep related impairment SF	PROMIS GLOBAL Physical Health − 0.69*	− 0.10	− 0.51*
PROMIS fatigue SF	PROMIS GLOBAL Physical Health − 0.73*	0.10	− 0.46*
Caregivers of persons with HCT (n = 30)			
*Caregiver-specific HRQOL*			
TBI-CareQOL caregiver-specific anxiety	CAS Burden − 0.84*	− 0.38*	− 0.05
TBI-CareQOL caregiver strain	CAS Burden − 0.77*	− 0.20	− 0.14
*Mental HRQOL*			
Neuro-QoL anxiety SF	PROMIS GLOBAL Mental Health − 0.50*	− 0.08	0.06
Neuro-QoL depression SF	PROMIS GLOBAL Mental Health − 0.42*	− 0.17	− 0.03
PROMIS anger SF	PROMIS GLOBAL Mental Health − 0.47*	− 0.10	− 0.19
Neuro-QoL positive affect and well being SF	PROMIS GLOBAL Mental Health 0.77*	0.43*	− 0.11
NIH toolbox self-efficacy SF	Neuro-QoL Positive Affect and Well-Being 0.72*	0.07	0.07
NIH toolbox perceived stress SF	CAS Burden − 0.78*	− 0.27	0.03
*Social HRQOL*			
Neuro-QoL ability to participate in SRA SF	CAS Burden 0.65*	0.24	0.29
*Physical HRQOL*			
PROMIS sleep related impairment SF	PROMIS GLOBAL Physical Health − 0.50*	− 0.12	− 0.29
PROMIS fatigue SF	PROMIS GLOBAL Physical Health − 0.60*	− 0.24	− 0.13
Caregivers of Persons with Traumatic Brain Injury (n = 139)			
*Caregiver-specific HRQOL*			
TBI-CareQOL caregiver-specific anxiety	CAS Burden − 0.73*	− 0.05	− 0.20*
TBI-CareQOL caregiver strain	CAS Burden − 0.73*	− 0.02	− 0.30*
*Mental HRQOL*			
Neuro-QoL anxiety SF	PROMIS GLOBAL Mental Health − 0.64*	0.09	− 0.26*
Neuro-QoL depression SF	PROMIS GLOBAL Mental Health − 0.62	− 0.03	− 0.29
PROMIS anger SF	PROMIS GLOBAL Mental Health − 0.63*	− 0.03	− 0.26*
Neuro-QoL positive affect and well being SF	PROMIS GLOBAL Mental Health 0.75*	0.03	0.22*
NIH toolbox self-efficacy SF	Neuro-QoL Positive Affect and Well-Being 0.50*	− 0.07	0.24*
NIH toolbox perceived stress SF	CAS Burden − 0.51*	0.02	− 0.26*
*Social HRQOL*			
Neuro-QoL ability to participate in SRA SF	CAS Burden 0.64*	− 0.03	0.21*
*Physical HRQOL*			
PROMIS sleep related impairment SF	PROMIS GLOBAL Physical Health − 0.52*	− 0.01	− 0.22*
PROMIS fatigue SF	PROMIS GLOBAL Physical Health − 0.57*	0.03	− 0.22*

*PROMIS* Patient Reported Outcomes Measurement Information System; *Neuro-QoL* Quality of Life in Neurological Disorders; *TBI-CareQOL* Traumatic Brain Injury Caregiver Quality of Life; *NIH* National Institutes of Health Toolbox, *CAS* Caregiver Appraisal Scale, *SF* = Short Form

*p > 0.05

## Discussion

This report provides data to support the reliability and validity of scores on several HRQOL measures from the TBI-CareQOL measurement system (which was developed specifically for use in caregivers of people with TBI) in an independent sample of caregivers of people with TBI, as well as in three new caregiver populations: caregivers of people with SCI, caregivers of people with Huntington disease, and caregivers of people with cancer. Measurement validation is an ongoing process, and data are needed across multiple studies and multiple samples over time. These data provide the beginning of what will hopefully eventually become a large body of evidence to support the clinical utility of these measures across diverse caregiver populations. No individual finding (that either supports or refutes a priori hypotheses for different psychometric properties) is sufficient for establishing the clinical utility of a new measure, rather it is the cumulative body of evidence that provides support for the reliability and validity of the scores in any given population [75]. As is evident in this sample, measurement properties will vary by cohort, as well as by study, and an understanding of measurement properties, and of the strengths and weaknesses of a particular measure in a particular population, is critical to proper score interpretation.

In this report, the psychometric properties were the strongest (i.e., most consistent with our a priori hypotheses/criteria) for the cohort of caregivers with TBI, which was also, subsequently, the largest cohort in this report. This was not especially surprising given: (1) many of the TBI-CareQOL measures were developed and calibrated in caregivers of TBI, and (2) that the sample of this cohort was more than quadruple that of the other cohorts (larger samples result in more stable score estimates). There is almost always a loss of sensitivity and specificity the “further away” you get from the measurement development cohort. This loss can be greater or smaller depending on both the heterogeneity of the initial development sample, and the number of characteristics that the development cohort has in common with the new cohort. This issue is at the very heart of many debates about the clinical utility of generic measures (i.e., measures that can be used across populations and allow for cross-disease comparison), versus specific measures (i.e., measures developed specifically for a given population that include content that may or may not be relevant to other groups). Yet, it is also important to note that even though the sample sizes of the other three cohorts was substantially smaller, the pattern of findings is largely identical which is supportive of the clinical utility of the TBI-CareQOL measurement system more broadly across caregiver populations.

With regard to the individual measures and the subgroup analyses, there was consistent support for both the reliability and validity of the scores on these HRQOL measures for the three new caregiver cohorts. For all HRQOL measures and all four caregiver subgroups, internal consistency reliability was supported. For caregivers of people with SCI, two measures indicated acceptable reliability (depression and self-efficacy) whereas all other measures had either good or excellent reliability. For caregivers of people with HCT, there were also two measures with acceptable reliability (depression, as also seen in SCI, but also caregiver-specific anxiety); again, all other measures had either good or excellent reliability. For both HD and TBI all measures were in the good to excellent range.

With regard to other measurement properties, none of the measures had ceiling effects, and only a few had floor effects (and most of these were pretty small). The measures with evidence of a floor effect were specific to mental health concepts: specifically, for anxiety (there was a minimal effect for caregiver-specific anxiety and a slightly more substantial floor effect for anxiety in SCI) and depression (there was a substantial floor effect in SCI, HD, and HCT). Given that most clinical or research interventions are focused on mitigating negative symptoms (such as depression and anxiety) a floor effect is generally not especially problematic given that most interventions are focused on reducing symptoms when they are present, or on detecting people that may be experiencing clinically significant problems. Those individuals, at the floor, are not the focus of such inquiries.

In addition, all TBI-CareQOL measures had moderate to strong relationships with comparator measures across all the caregiver cohorts. In general, almost all of the hypothesized discriminant validity comparators (caregiver ideology and caregiver mastery) had small to negligible relationships with the TBI-CareQOL HRQOL measures. There were a couple of exceptions to our hypotheses: there was a moderate relationship between caregiver-specific anxiety and caregiver ideology for caregivers of people with SCI. There was also a moderate relationship between caregiver strain and caregiver ideology for caregivers of people with HD. While we did not anticipate these relationships, retrospective analysis of the literature provides some possible explanation. For example, even though there were very few significant differences among the groups for the different demographic variables, it is possible that the observed trends for demographic differences among the groups may partially explain these unanticipated findings. For example, the SCI caregivers reported the longest time in the caregiver role relative to the other groups and therefore may also have stronger ratings of caregiver ideology. Similarly, the HD group had the highest number of male caregivers and had the highest percentage of being single/divorced, the highest percentage being employed full-time, the oldest caregivers, and the highest percentage of spousal caregivers, which are all factors that could be associated with worse caregiver strain. In addition, caregivers who hold traditional ideologies, such as perceiving caregiving as a family obligation, may be less likely to experience strain and/or anxiety regarding the caregiver role. Prior research has demonstrated that a perception of benefit from providing support to others, including family members, is associated with lower mortality [76] and less depression [77]. Thus, it is also possible that caregiver ideology reflects underlying resilience, which has been found to impact risk of stress or burden for caregivers [78].

Study limitations include the secondary use of data from a randomized control trial that was not designed specifically to establish the psychometric properties of these new measures. In addition, the small sample size of the three new caregiver cohorts (SCI, HD, and HCT) precluded our ability to examine known groups validity, or responsiveness to change. Future work is needed to examine these additional indices of validity in these new cohorts. In addition, although rates for race/ethnicity in the HD cohort were consistent with established prevalence rates (i.e., HD is considered a euro-ethnic disease) [79–82] the absence of any racial or ethnic diversity in this group limits the generalizability of these findings.

## Conclusion

Given that this sample represents four distinct caregiver cohorts—two cohorts of caregivers of people that have an injury caused by a traumatic event (either TBI or SCI), a cohort of caregivers of persons with a fatal neurodegenerative disease, and a cohort of caregivers of people with a relapsing and remitting cancer condition—these data would also suggest that these measures might also be extended for use in other caregiver cohorts with similar etiology/presentation (e.g., caregivers of people with Alzheimer’s disease, stroke, Parkinson’s disease, multiple sclerosis, or other cancer conditions) and used more broadly in the caregiver literature. While this does not preclude the need for additional reliability and validity data in new caregiver cohorts, they do provide evidence that would suggest a pattern of findings that is likely generalizable more broadly across caregiver groups beyond TBI, SCI, HD, and HCT.

## Data Availability

NCT04556591: Participant data collected during the trial, after de-identification, will be available for sharing with individuals in the scientific community, upon request from the study PI (Noelle E Carlozzi; PMR-CODAlab@med.umich.edu). The data is currently available. An institutional data use agreement will be required before data is shared. NCT04570930: Participant data collected during the trial, after de-identification, will be available for sharing with individuals in the scientific community, upon request from the study PI (Noelle E Carlozzi; PMR-CODAlab@med.umich.edu). The data will be available after the main findings are published; data is expected to be available in late 2024/early 2025. An institutional data use agreement will be required before data is shared.

## Abbreviations

HCT: Allogeneic hematopoietic cell transplantation
HD: Huntington disease
HRQOL: Health-related quality of life
NIH: National Institutes of Health
PROMIS: Patient-Reported Outcome Measurement Information System
SCI: Spinal cord injury
